# Recent Progress on Cellulose-Based Electro-Active Paper, Its Hybrid Nanocomposites and Applications

**DOI:** 10.3390/s16081172

**Published:** 2016-07-26

**Authors:** Asif Khan, Zafar Abas, Heung Soo Kim, Jaehwan Kim

**Affiliations:** 1 Department of Mechanical, Robotics and Energy Engineering, Dongguk University-Seoul, 30 Pildong-ro 1-gil, Jung-gu, Seoul 04620, Korea; khanuet11@gmail.com (A.K.); zafar_sial229@hotmail.com (Z.A.); 2 Center for EAPap Actuator, Department of Mechanical Engineering, Inha University, 253 Yonghyun-dong, Nam-Gu, Incheon 22212, Korea; jaehwan@inha.ac.kr

**Keywords:** electro-active paper, polymers, cellulose, transducers, nanocomposite

## Abstract

We report on the recent progress and development of research into cellulose-based electro-active paper for bending actuators, bioelectronics devices, and electromechanical transducers. The cellulose electro-active paper is characterized in terms of its biodegradability, chirality, ample chemically modifying capacity, light weight, actuation capability, and ability to form hybrid nanocomposites. The mechanical, electrical, and chemical characterizations of the cellulose-based electro-active paper and its hybrid composites such as blends or coatings with synthetic polymers, biopolymers, carbon nanotubes, chitosan, and metal oxides, are explained. In addition, the integration of cellulose electro-active paper is highlighted to form various functional devices including but not limited to bending actuators, flexible speaker, strain sensors, energy harvesting transducers, biosensors, chemical sensors and transistors for electronic applications. The frontiers in cellulose paper devices are reviewed together with the strategies and perspectives of cellulose electro-active paper and cellulose nanocomposite research and applications.

## 1. Introduction

Cellulose, with a yearly estimated biomass production of 1.5 trillion tons, is an almost inexhaustible polymer raw material with a fascinating structure and properties [1]. Since cellulose is biodegradable, renewable and biocompatible, its derivatives are used for many applications including the immobilization of proteins and antibodies, coatings, laminates, optical films, pharmaceuticals, textiles, foodstuffs, as well as the formation of cellulose composites with synthetic polymers and biopolymers. The dipolar orientation and trapped charge are the two major phenomena that contribute to the pyro-, piezo-, and ferro-electricity of polymers. Shear piezoelectricity in polymers of biological origin such as cellulose and collagen was discovered in the 1950s [2,3]. Piezoelectricity was observed in the uniaxially oriented systems of crystallites of cellulose and elongated films of optical synthetic polymers. The shear piezoelectricity in cellulose based biopolymers, such as wood, ramie, chitin, amylase and starch, is comparable to that in quartz crystal [4]. The discovery of tensile piezoelectricity in stretched polyvinylidene fluoride (PVDF) triggered research into PVDF and its copolymers in subsequent years [5]. Shear piezoelectricity in wood depends largely on the type of wood, its orientation, and the environmental conditions. Despite these early inroads, there have been very few investigations into the potential of cellulose to be used as a smart material until Kim discovered an interesting actuation mechanism in the cellulose paper. This smart cellulose was named electro-active paper (EAPap) [6]. When an electric voltage was applied to the electrodes, EAPap produced a bending displacement depending on the actuation voltage, frequency, host paper type, and adhesive. To improve the performance of the EAPap actuator, the coating of a conducting polypyrrole onto the cellulose film provided good results for actuators using microwave energy [7,8]. The working principle of cellulose EAPap is claimed to be a combination of the piezoelectric effect and ionic migration effects, associated with the dipole moment of the cellulose paper ingredients. After cellulose-based EAPap has satisfied the requirements of a smart material, various applications are possible including, wirelessly controlled EAPap actuators, flexible speakers, flying objects, paper transistors and MEMS/NEMS devices [9,10,11,12,13]. The use of single walled carbon nanotube (SWNT)/multi-walled carbon nanotube (MWNT)-cellulose hybrid actuators improves the performance in terms of the force and actuation frequency [14]. The coating of MWNTs on cellulose EAPap overcomes the drawbacks of the low output force and low actuation frequency [12]. Cellulose and chitosan blend EAPap was introduced to solve the issue of degradation with time and sensitivity to humidity that affects the performance of EAPap actuators. The electromechanical coupling and mechanical properties of cellulose EAPap have been reported to be very similar to those of piezopolymers [15]. This extends its possible uses to strain sensors, self-powered vibration sensors, and energy scavenging transducers [16]. Besides, natural cellulose, in particular nanocrystalline or nanofibrillated cellulose, has unique properties, including a high Young’s modulus, dimensional stability, low thermal expansion coefficient, outstanding reinforcing potential and transparency [17,18]. Bacterial cellulose composites are unique and promising for medical implants and scaffolds in tissue engineering [19,20,21]. The use of hybrid cellulose nanocomposites with enhanced material properties has been studied for biosensors, disposable chemical sensors, energy conversion and various other applications [22,23,24,25,26]. With the addition of a metal oxide to the cellulose matrix, the chemical stability, mechanical properties, conductivity and photosensitivity can be enhanced, thereby allowing cellulose to be used in bioelectronics applications [27].

In this paper, we review the recent advances in cellulose electro-active paper and its hybrid nanocomposites. The actuation mechanism, physical properties and electromechanical behavior, including the piezoelectricity of cellulose EAPap, are briefly summarized, and cellulose-based hybrid nanocomposites are introduced in terms of hybridizing carbon nanotubes, polymer coatings, and chitosan blends, as well as metal oxides with a cellulose substrate. The recent progress and development of cellulose-based bending actuators, vibration sensors, biosensors, and disposable chemical sensors, as well as their applications in acoustic and bioelectronics, are critically discussed. Finally, the possibilities and challenges in the area of cellulose EAPap and its hybrid composites are addressed.

## 2. Cellulose EAPap

### 2.1. Actuation Mechanism

**Figure 1:**
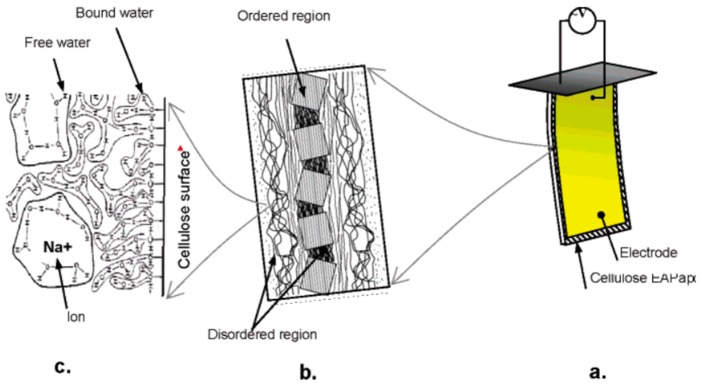
Concept of electro-active paper actuator: (**a**) EAPap is made from cellulose paper on which gold electrodes are deposited on both sides; (**b**) cellulose microfibril has ordered crystalline regions and disordered regions; (**c**) water molecules are bonded with hydroxyls on the cellulose surface (bound water) or clustered in free (free water) [28].

Considering the structure of cellulose and processing of cellulose-based EAPap, Kim et al. [28] believed that the actuation of EAPap is due to a combination of two mechanisms: ion migration and dipolar orientation. Cellulose EAPap material is a sheet of regenerated cellulose and, morphologically, regenerated cellulose has ordered and disordered regions. The ordered domains are mostly crystalline, and the disordered molecules retain their preferential direction parallel to the chains in the microfibrils and form surface disorder on the microfibrils. The conceptual configuration of cellulose EAPap is depicted in Figure 1a. The concept of the microfibril is shown in Figure 1b. The EAPap material has large regions of disordered cellulose chains, where water molecules can be found attached to hydroxyl groups (Figure 1c). During the fabrication of cellulose paper, sodium ions are injected into the paper fiber. When an external electric field is applied, these ions become mobile and can migrate to the anode. In addition, the molecular motion of free water in the disordered region is not restricted by the cellulose molecules, and the water molecules can interact with the ions in the cellulose. In the presence of an electric field, the sodium ions surrounded by free water molecules move to the anode. The selective ionic and water transport across the polymer under an electric field results in volumetric changes, which in turn lead to bending. When a dc electric field was applied, the cellulose EAPap actuator was bent toward the positive electrode, which confirmed the actuation mechanism. The attractive actuation mechanism of cellulose EAPap renders many possible applications, such as sensors, actuators, flexible speakers, self-powered vibration sensors and flying objects. The addition of poly(ethylene oxide)-poly(ethylene glycol) (PEO-PEG) affects the actuation behavior of cellulose electro active paper, resulting in a maximum bending displacement of 5.0 mm with a very low electrical power consumption (7 mW/mm) under ambient conditions.

The increased displacement output and decreased electrical power consumption has two distinct elastic constants. The modulus of EAPap is dependent on the orientation of the specimen of the actuator might be due to the improved polymer chain flexibility and ion mobility. The ion migration effect might play a more important role in the actuation principle [29].

### 2.2. Mechanical Properties

**Figure 2:**
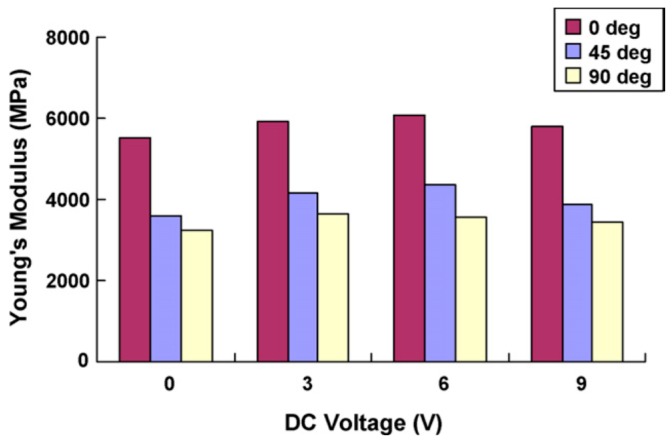
Variation of elastic modulus with electric excitation [31].

**Figure 3:**
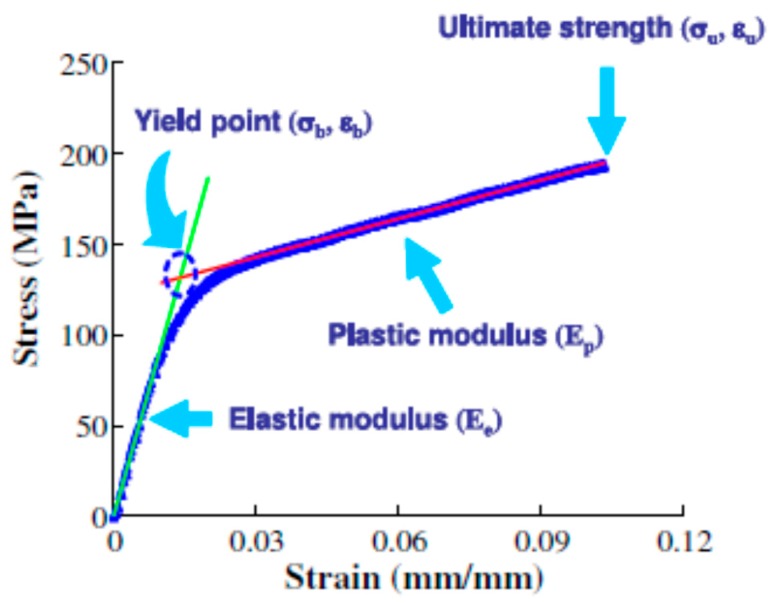
Typical pull test results of cellulose EAPap [32].

EAPap is a complex anisotropic material, and material testing confirmed that EAPap has two distinct elastic constants. The modulus of EAPap is dependent on the orientation of the specimen. Linear creep behavior was also observed by applying a constant stress and low frequency cycling loading [30]. This out-of-plane bending deformation of EAPap is good for achieving flapping wings, micro-insect robots, and smart wallpapers. On the other hand, in-plane strains, such as the contraction and extension of EAPap materials, are also promising for artificial muscle applications, due to the high value of their Young’s modulus [31]. The initial estimate of the Young’s modulus of the 45° EAPap sample orientation was 5.5 GPa (see Figure 2). This in-plane strain of cellulose EAPap can be useful for artificial muscle applications. Kim et al. [32] made a special material testing setup to investigate the material properties under different environmental conditions such as temperature and humidity. The initial Young’s modulus of EAPap was in the range of 4–9 GPa, which was higher than that of other polymer materials. The two Young’s moduli observed may be associated with the microfibrils and amorphous regions of cellulose. The elastic strength and stiffness decreased with increasing humidity and temperature. A typical stress-strain curve obtained from the pull test of cellulose EAPap is presented in Figure 3.

A hygrothermal behavior study under coupled temperature and humidity conditions revealed that increased humidity did not have a significant impact on the creep deformation. The EAPap performance at room temperature is better than at elevated temperature [33]. Kim et al. [34] described the elastic, viscous and creep characteristics of cellulose Electro-Active Paper employing a mechanical model.

EAPap creeps at elevated temperature, but there is a little creep at room temperature. The elastic behavior varies inversely with the temperature and the bias angle; however, the overall viscous behavior of the three types of EAPap tested is relatively complex. The micro-scale creep deformation is responsible for the changes in the EAPap structure [35]. The effects of heat treatment, Li^+^ ions, and solvent mixture on the structure, performance, properties, piezoelectricity, and actuation behavior have also been reported [36,37,38,39].

### 2.3. Electromechanical Behavior and Direct Piezoelectricity

**Figure 4:**
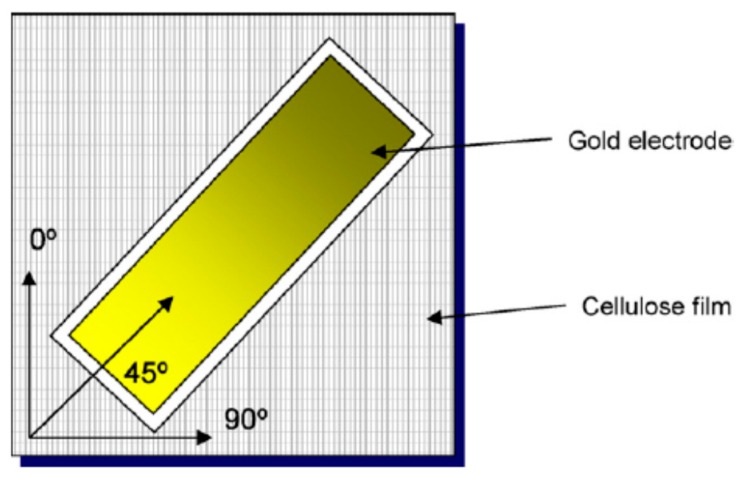
Orientation of cellulose film and schematic of EAPap [40].

The actuation mechanism of EAPap is a combination of ion migration and the piezoelectric effect. Cellulose chains are known to form hydrogen bonding, leading to the formation of microfibrils with partially crystalline parts, making cellulose a semicrystalline polymer. Hydrogen bonding contributes to the dipolar orientation by stabilizing the dipoles and leading to a permanent polarization that results in piezoelectric behavior. Kim et al. [40] investigated the electromechanical coupling by applying an electric field when the cellulose based EAPap was mechanically strained. The piezoelectric charge constants of three different orientation EAPap samples under quasi-static direct piezoelectricity were measured as shown in Figure 4.

The measured piezoelectric charge constant of the 45° sample was 28.2 pC/N, which was larger than those of the 0° and 90° samples. The measured piezoelectric charge constant of EAPap is similar to those of piezo polymers such as PVDF. Yang et al. [15] suggested the use of the wet drawing process to align the cellulose fibers to increase the piezoelectricity of EAPap. The crystallinity and alignment of the cellulose film, as well as its stiffness, was increased by increasing the drawing ratio. The highest value of the piezoelectric charge constant measured was 27.3 pC/N at a drawing ratio of 2.0 for the 45° aligned sample.

This strong shear piezoelectricity of EAPap can be advantageous to develop potential applications such as strain sensors, microphones, and film type flexible speakers. The electrically aligned regenerated cellulose films showed a higher in-plane piezoelectric constant due to the increased crystallinity index [41]. The heat treatment of the EAPap specimen also improved the piezoelectric effect as compared to the specimen with no heat treatment [36]. To understand the dielectric behavior of EAPap as a novel piezoelectric material, Yun et al. [42] revealed that the dielectric constant of EAPap was frequency and temperature dependent. The largest change in the dielectric constant was observed at 0 °C, while the highest dielectric constant was obtained at around 100 °C, which might be related to the dipolar behavior of the hydroxyl structure of cellulose and absorbed or existing internal water molecules in cellulose EAPap.

## 3. Cellulose Hybrid Nanocomposites

### 3.1. Cellulose-SWNT/MWNT EAPap

**Figure 5:**
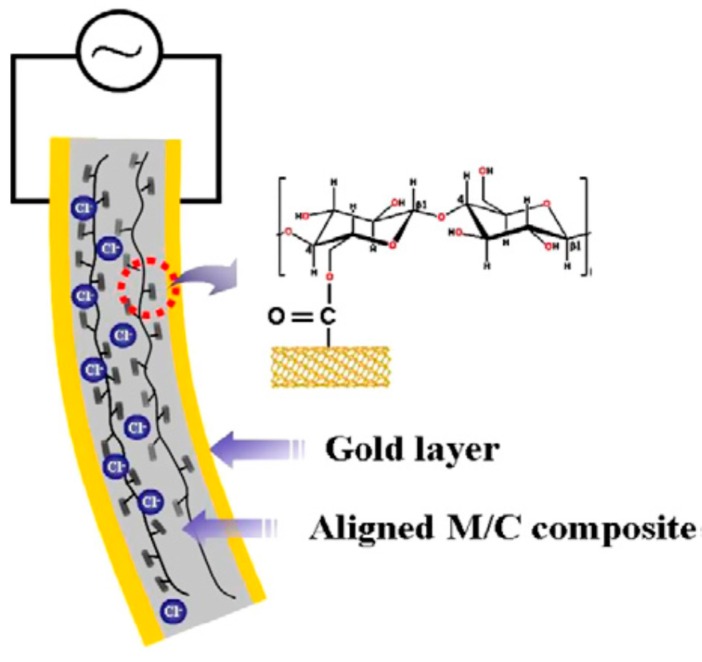
Schematic structure of aligned M/C composite actuator [48].

Biodegradability, low power consumption, low actuation voltage, large displacement, light weight and dryness are some of the main advantages of EAPap materials. However, improvements in frequency band, mechanical force output and stiffness of the EAPap material are essential. Whereas, carbon nanotubes possess excellent mechanical, thermal and electrical properties. CNT/cellulose composites have been studied to obtain positive effects of each promising material, by the incorporation of CNT and polymers [43,44]. Therefore, the synergy between EAPap and the multifunctionality of CNTs may not only improve the characteristics of the existing EAPap materials, but also broaden their application possibilities. To improve the actuation force and frequency bandwidth of EAPap actuators, a conducting polymer and carbon nanotube (CNT) coated cellulose based electro-active paper hybrid actuator was characterized [45]. Yun et al. [46] tested the performance of a bending electro-active paper actuator made by mixing MWNTs and cellulose in terms of its Young’s modulus, mechanical power output, and resonance frequency. Functionalized multi-walled carbon nanotubes (F-MWNTs) were blended with the cellulose solution to fabricate F-WNTs/cellulose EAPap actuators, resulting in a large bending displacement of 4.5 mm and improved output force [47]. The mechanical and electrical properties and piezoelectric behavior of aligned MWNT/cellulose composites formed by a stretching process were also demonstrated by observing their morphology, properties, stretching ratios and humidity level (Figure 5) [48].

The aligned MWCNTs enhanced the mechanical and piezoelectric properties, as well as the performance, of the CNT/Cellulose composite. Other notable cellulose-carbon nanotubes hybrid nanocomposites characterized were titanium dioxide (TiO_2_)/MWNT/cellulose hybrid nanocomposites for pH sensors [49], biocompatible composites of cellulose and carbon nanotubes for cell sensors [50], and aligned single-walled carbon nanotube bonded cellulose composites for flexible paper transistors [51]. Pankonian et al [52] studied SWNT-cellulose nanofiber composites formed by the electro spinning technique for smart applications.

### 3.2. Cellulose-Chitosan Blended EAPap

**Figure 6:**
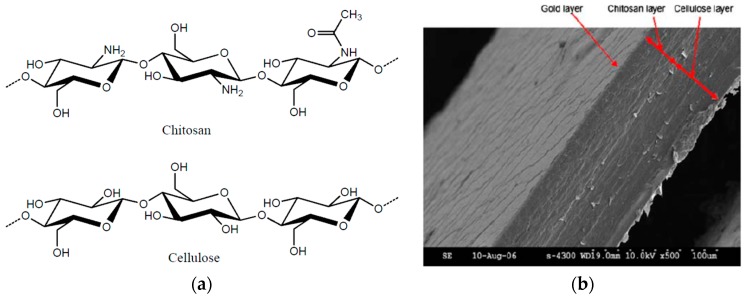
(**a**) Chemical structures of chitosan and cellulose [58]; (**b**) Scanning electron microscope image at the cross section of the laminated film [55].

Chitosan is a biocompatible polymer that exhibits a number of functional biological properties and cellulose-chitosan nanocomposites exhibit enhanced mechanical properties [53,54]. Due to the compatible nature of cellulose and chitosan, blending chitosan with cellulose can help to endow an actuator with superior characteristics. The effect of the chitosan concentration on the electrical properties and different types of free ions was studied to investigate the actuation behavior of cellulose-chitosan laminated films [55,56]. Figure 6 depicts the structure of cellulose and chitosan and an SEM image at the cross section of the laminated film. Wang et al. [57] examined the effects of chitosan and acetic acid on the actuation behavior of the EAPap actuator, and the optimum mole ratio of the acetic acid and chitosan structure unit was found.

Cellulose EAPap is very sensitive to humidity and temperature, and its electromechanical behavior degrades with time. Cai and Kim [59] tried to diagnose these pitfalls by cracking an EAPap actuator made with cellulose and chitosan blends. The results revealed that the chitosan-cellulose-based EAPap actuator is less sensitive to humidity; a large bending displacement of about 4.1 mm and long lifetime were observed. The performance of the inter-penetrated network (IPN) based chitosan-cellulose actuator was evaluated in terms of the bending displacement with respect to the actuation frequency, voltage, humidity level, chitosan content, and time.

It was observed that as the chitosan content in the IPNs increased, the crystallinity decreased and the network became denser, which caused the Young’s modulus to increase [60]. With increasing chitosan blend ratio, the dielectric loss factor decreased, while the real part of the dielectric constant was increased. Jang et al. [61] presented a theoretical model for the bending displacement of a (chitosan blended cellulose)-EAPap actuator and confirmed the results with experimental verification. Due to the better performance and electromechanical behavior of chitosan-blended cellulose, chitosan-cellulose EAPap is promising for many biomimetic applications in the foreseeable future, including micro-robots, artificial muscles, and various other actuators.

### 3.3. Metal Oxide Cellulose Nanocomposites

**Figure 7:**
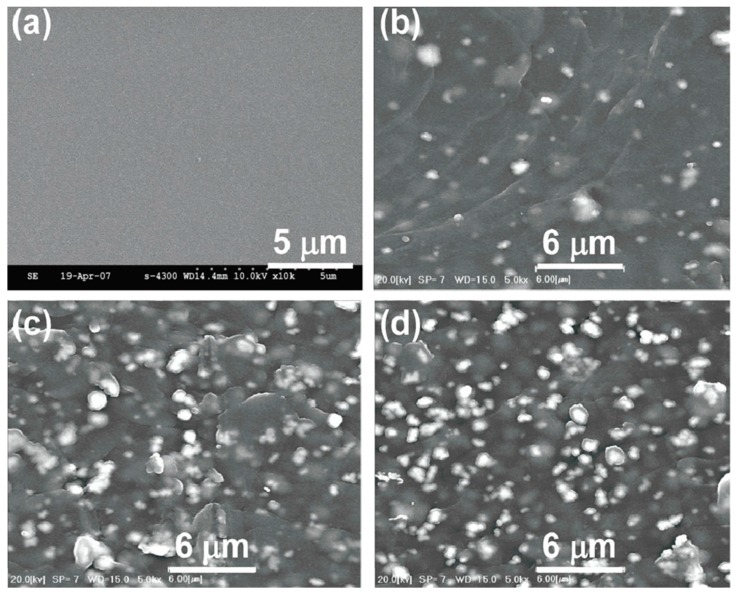
Surface SEM images of cellulose (**a**) and SnO_2_-cellulose hybrid thin films as a function of SnO_2_ concentration: 10 wt % (**b**) 20 wt % (**c**) and 30 wt % (**d**) [70].

The material properties, including the chemical stability, conductivity, photosensitivity, and mechanical properties of cellulose for sensor and electronics applications can be enhanced by adding metal oxides to the cellulose matrix [27,62,63,64,65,66]. This hybrid characterization of cellulose based nanocomposites has broadened the applications area of cellulose. Yadav et al. [67] prepared a high performance iron oxide/cellulose nanocomposite film by impregnation of iron oxide nanoparticles into regenerated cellulose film. The tensile strength and elastic modulus were improved by 39% and 57%, respectively, compared with the regenerated cellulose. Moreover, the iron oxide/cellulose nanocomposite was thermally more stable than the regenerated cellulose film. Costa et al. [68] introduced a cellulose derivative composite for electro-optical sensors. Marques et al. [69] prepared a TiO_2_-cellulose nanocomposite through titanyl sulfate hydrolysis in an acidic medium in the presence of cellulosic fibers. Optical studies revealed a much higher opacity for the synthetic sample. By hybridizing functional metal oxides with cellulose substrates, nature friendly, cost effective and disposable sensor devices with good sensing performance are achievable. Mahadeva et al. [70] reported on the preparation and characterization of a hybrid thin film consisting of tin oxide nanoparticles and cellulose as shown in Figure 7.

The reduction in the crystalline melting transition temperature due to the SnO_2_ nanoparticles showed the potential application of this hybrid configuration as a biodegradable and flexible humidity sensor. A cellulose nanocomposite with a high content of Fe_3_O_4_ nanoparticles with regenerated cellulose as a matrix provided a green and facile method for the preparation of bio-based nanocomposite films with excellent magnetic properties [71]. Mahadeva et al. [72] developed hybrid piezoelectric paper through fiber functionalization by anchoring nanostructured BaTiO_3_ into a stable matrix with wood cellulose fibers prior to the process of making paper sheets. The paper showed the largest piezoelectric coefficient, *d*_33_ = 4.8 ± 0.4 pC N^−1^, at the highest nanoparticle loading of 48 wt % BaTiO_3_. The strength of the piezoelectric hybrid paper decreased with increasing nanoparticle loading, whereas the response of the hybrid paper significantly increased with BaTiO_3_ content in the paper.

Kim et al. [63] investigated the optical, electrical and mechanical properties of one dimensional cellulose/silica and silica-gold hybrid biomaterials for various electronic applications. The electromechanical behavior of the green cellulose-ZnO hybrid nanocomposite showed superior piezoelectricity in longitudinal modes, with a value of 160 pC/N. However, bending mode is not efficient to utilize the piezoelectric effect of this green nanocomposite [73]. Research and development of the hybrid materials in conjunction with sensor devices are necessary to move forward to real applications in medical and bio-electronic applications. However, based on the modified current integration technology, cellulose based paper sensors have high potential to be used for cheap, disposable and biocompatible sensors, with recycling available after usage.

### 3.4 Cellulose-Graphene Nanocomposites

The exceptional properties of graphene and its derivatives, such as its Young’s modulus of 1 TPa, ultimate strength of 130 GPa and room-temperature electron mobility around 200,000 cm^2^·V^−1^·s^−1^ have attracted significant interests from researchers worldwide [74]. Nowadays, ploymers based on graphene and its derivatives have motivated several studies on cellulose/graphene composites. Kafy et al. [75] synthesized cellulose/graphene nanocomposite by grafting functionalized graphene oxide with cellulose. The Young’s modulus and dielectric properties were improved significantly compared with the pristine cellulose. The cellulose/graphene nanocomposite was found to be sensitive to organic solvents by capacitive change and could be used to distinguish various solvents based diffusion mechanism. The cellulose modified graphene oxide nanocomposite has been reported as an environmentally stable and excellent next generation material for flexible energy storage and electronics [76]. Sen et al. [77] loaded graphene nanoplatelets (0.10, 0.25 and 0.50 wt %) into a cellulose matrix to enhance the mechanical, electrical and electroactive performance of the cellulose based composite actuators. Increase in graphene loading enhanced the capability of the actuator operating at higher excitation voltage, but reduced the response rate of the actuator. A considerable increase in the electrical conductivity was reported by loading graphene into the cellulose film. The tensile strength and Young’s modulus increased with increasing graphene loading up to 0.25 wt %. Kim and his coworkers [78] reported a high fidelity bioelectronic soft actuator (named as TOBC-IL-G actuator based on based on biofriendly 2,2,6,6-tetramethylpiperidine-1-oxyl radical-oxidized bacterial cellulose (TOBC), chemically modified graphene, and ionic liquid [EMIM][BF_4_] as plasticizer. The developed TOBC-IL-G muscular actuator showed exceptionally large static deformation without apparent back-relaxation, much faster response time and highly durable harmonic actuation compared with the conventional biopolymer actuators. Ozdemir et al. [79] investigated the effects of graphene loading (0.1, 0.2, 0.3 wt %) on both the electromechanical and mechanical properties of carboxymethyl-cellulose (CMC)-based actuators. The ultimate tensile strength of CMC-based actuators containing 0.3 wt % graphene was higher than that of unloaded actuators by approximately 72.8%. Furthermore, increasing the graphene content continuously increased the value of Young’s modulus of the graphene loaded actuators. Wang and his coworkers [80] demonstrated a water-based method to fabricate strong, electrically and thermally conductive hybrid thin films (papers) made from the combination of graphene nanoplatelets (GnP) and cellulose nanocrystals (CNC). The mechanical properties of the hybrid GnP papers were improved greatly by the incorporation of CNC, and the hot-press process further enhances the mechanical properties by eliminating internal pores and forming better particle alignment.

## 4. Applications

### 4.1. Cellulose EAPap Actuators

**Figure 8:**
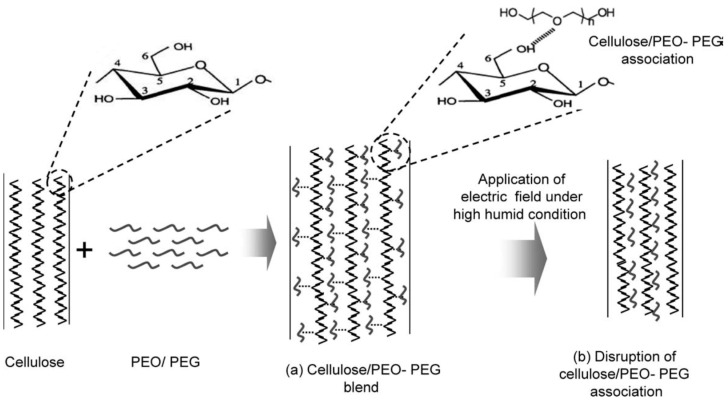
Schematic representation of association of PEO-PEG with cellulose and its disruption under the excitation of an electric field under high humidity conditions [86].

Cellulose-based electro-active paper (EAPap) has been reported as a smart material with various advantages such as its light weight, large displacement output, sustainable, and low actuation voltage. However, the performance of EAPap actuators is very dependent on the environmental conditions and, in particular, it requires high humidity [81]. To develop an EAPap actuator that is less sensitive to humidity, Wang et al. [82] evaluated the performance of an EAPap bending actuator made with LiCl/cellulose films. The displacement output was increased at room condition humidity, which confirms the possibility to develop actuators that are less sensitive to humidity. The actuator made by the electrochemical deposition of conductive polyaniline on a cellulose paper showed threefold better performance as compared to the EAPap actuator without a conductive coating [83]. Kim et al. [84] reported that EAPap made with cellulose and sodium alginate produces its maximum displacement at a lower humidity level than a simple cellulose paper actuator. The effects and optimum values of the glycerol content and the thickness effects were also studied on a gold electrode deposited EAPap actuator [85]. The effects of the humidity and polyethylene oxide (PEO)-polyethylene glycol (PEG) content on the performance of the cellulose/PEO-PEG microcomposite actuator showed that the maximum bending displacement of the actuator was nearly twice that of the cellulose EAPap actuator when the PEO-PEG content was 5% [86,87]. However, further increasing the PEO–PEG content resulted in decreased actuator performance, which might be due to the increased intermolecular interaction caused by hydrogen bonding that reduces the mobility of the molecules. The schematic representation of association of PEO-PEG with cellulose in illustrated in Figure 8.

To study the effects of the electrode pattern on the performance of the actuator, EAPap actuators with fishbone and regular rectangular pattern electrodes were fabricated, and their performance was examined in terms of the frequency and bending displacement [88]. Mechanical stretching and the alignment of the cellulose film by electrospinning also increased the performance and electromechanical efficiency of the EAPap actuator [89,90,91]. Tape casting and zone stretching automated processes were suggested for the mass production of EAPap actuators [92]. The mechanical output and efficiency of the actuator with the optimal thickness were drastically improved compared with the previous study results [93]. The blends of ionic liquid with cellulose showed better performance and more durable EAPap actuators that can solve the drawback of the performance of EAPap actuators at low humidity levels [94,95,96].

**Figure 9:**
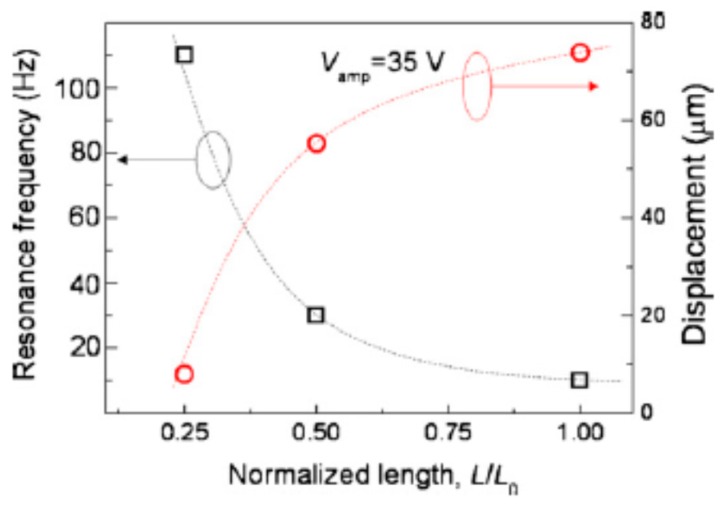
Comparison of the resonance frequency and the bending displacement as a function of the normalized length of the unimorph EAPap actuator for haptic applications [98].

Yun et al. [97] attempted to measure the blocked force of an EAPap actuator using a cantilever force transducer and concluded that the proposed technique can be used for AC as well as DC electric field excitation with high resolution in the µN range. Yun et al. [98] tested the performance of the EAPap actuator for tactile sensation. The experimental results confirmed that stacked and unimorph EAPap actuators can be used for haptic applications, due to their ability to stimulate Merkel’s disk and the Meissner corpuscle (see Figure 9).

A film type haptic actuator was made with two cellulose acetate active membranes and a comparison of its air gap performance with ones made of polyethylene terephthalate and polyvinyl chloride membranes showed that it exhibited superior displacement output due to its higher dielectric constant. The cellulose acetate double membrane actuator revealed the potential of kinesthetic actuators for haptic devices [99].

**Figure 10:**
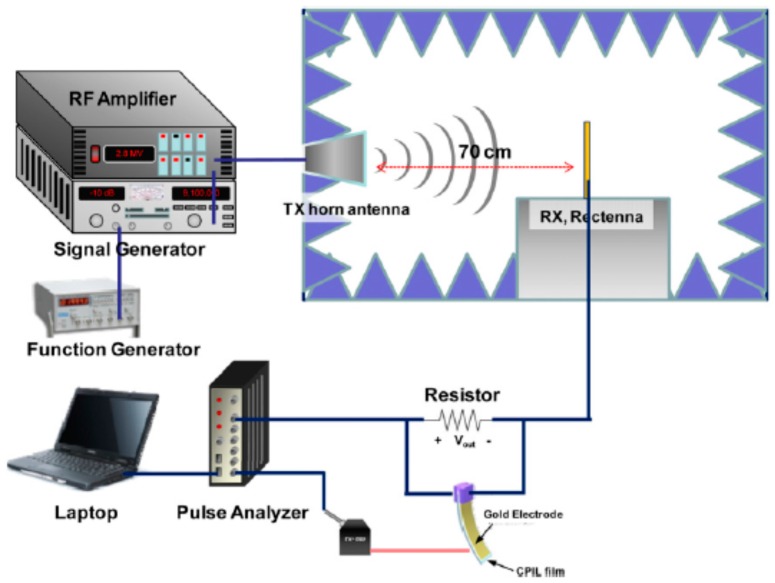
Schematic of microwave power transmission test setup [101].

**Figure 11:**
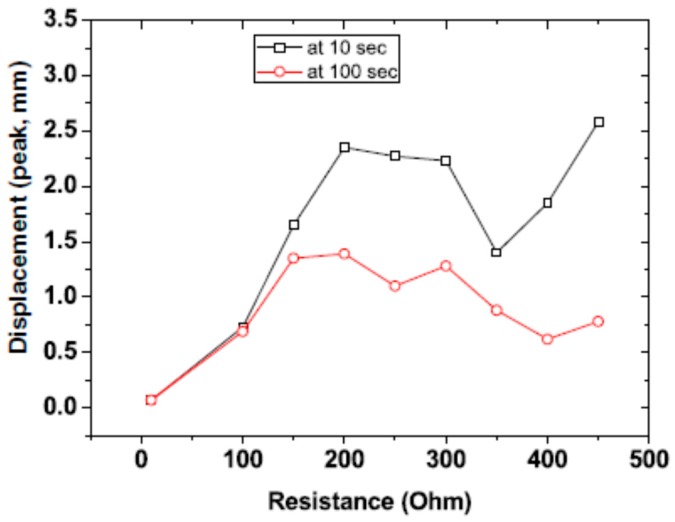
Effects of the charge dissipation resistance on the bending displacement of the CPIL-EAPap actuator: Bending displacements at 10 and 100 s [101].

Yang et al. [100] fabricated a wirelessly driven cellulose-polypyrrole-ionic liquid (CPIL) nanocomposite actuator by incorporating nanoscale polypyrrole (PPy) onto cellulose by an in situ polymerization technique followed by activation in a room temperature ionic liquid. The CPIL actuator showed a maximum bending displacement of 10 mm under ambient humidity conditions with an electrical power consumption of 30 mW. Yang et al. [101] proposed the concept of a remotely powered and controlled EAPap actuator by means of modulating waves with a control signal and demodulating the actuator through the rectenna (rectifying antenna) rectification. Figure 10 shows a schematic of the microwave power transmission setup. Without microwave modulation, the maximum voltage and power obtained from the dipole rectenna array were 11.4 V and 324 mW, respectively. With modulated microwaves, the maximum output voltage, and power were slightly increased to 12.8 V and 410 mW, respectively. Within the optimum resistance range, the CPIL actuator was successfully actuated with the modulated microwaves without an onboard controller, and a maximum displacement of 2.4 mm was obtained (Figure 11). Modulated microwave power transmission technology is very useful for remotely driven actuators, biomimetic robots, remote sensing units and portable electronics.

### 4.2. EAPap Vibration Transducers

The ability of electro-active paper vibration sensors to measure the dynamic characteristics of vibrating structures based on piezoelectricity reveals the vibration sensing capabilities of cellulose EAPap. Due to the bidirectional strain characteristics of EAPap, it’s bending modes as well as twisting modes resonant frequencies were accurately measured from the impulse response of the cantilever beam [16,102]. Kim et al. [103] investigated the potential of EAPap to be used for beam vibration control by attaching an EAPap sensor and actuator patches on a cantilever beam.

**Figure 12:**
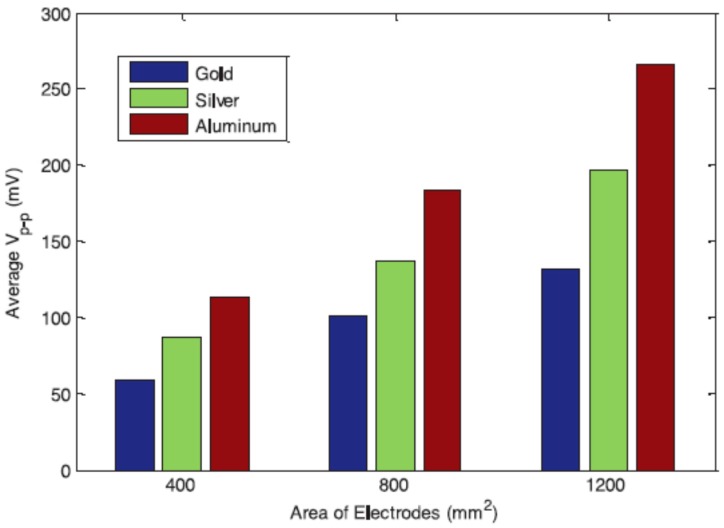
Average peak to peak open circuit voltage output for 400 mm^2^, 800 mm^2^, and 1200 mm^2^ gold, silver and aluminum electrodes coated on EAPap [108].

A PID controller was designed to suppress the vibration by considering the sensor output position error, and promising results were achieved in terms of the use of EAPap piezoelectric patch in vibration control. Alam and Mandal [104] presented a flexible hybrid piezoelectric generator (HPG) based on native cellulose microfiber (NCMF) and polydimethylsiloxane (PDMS) with multi-wall carbon nanotubes (MWCNTs) as conducting filler while avoiding further chemical treatment of the cellulose and traditional electrical poling steps for piezoelectric voltage generation. The HPG showed an open circuit output voltage of ~30 V and short circuit output current of ~500 nA, corresponding to a power density ~9.0 μW/cm^3^ under repeated hand punching. Abas et al. [105] tested the potential of cellulose EAPap coated with gold electrodes to be used as an electromechanical energy harvesting transducer. The use of a cellulose based energy harvesting transducer is in-line with the spirit of green technologies [106], and can be effective for harvesting energy from ambient vibrations like other piezoelectric materials [107]. The electrode area and material were the major factors to obtain the highest output voltage and corresponding power from an aluminum electrode coated EAPap electromechanical transducer as shown in Figure 12 [108].

### 4.3. Cellulose-Nanocomposite Biosensors

Polymer-based sensors have gained a great deal of attention in recent times, due to their flexibility and ability to collect molecules on their sensing surface. With the discovery of cellulose as a smart material, its use for paper based electronics devices such as actuators and sensors has been envisaged. Ren et al. [109] reported on an amperometric glucose biosensor based on a gold nanorods/cellulose acetate composite film as an immobilization matrix. Under optimal conditions, the biosensor showed high sensitivity (8.4 μA/cm^2^·mM), a low detection limit, and good storage stability. Esmaeili et al. [110] investigated a glucose biosensor by integrating polypyrrole-cellulose nanocrystal-based composites with glucose oxidase (GOx). The developed biosensor exhibited a high sensitivity (ca. 0.73 μA·mM^−1^), with a high dynamic response ranging from 1.0 to 20 mM glucose and a limit of detection (LOD) of (50 ± 10) μM. The sensor showed an acceptable reproducibility and stability over time.

**Figure 13:**
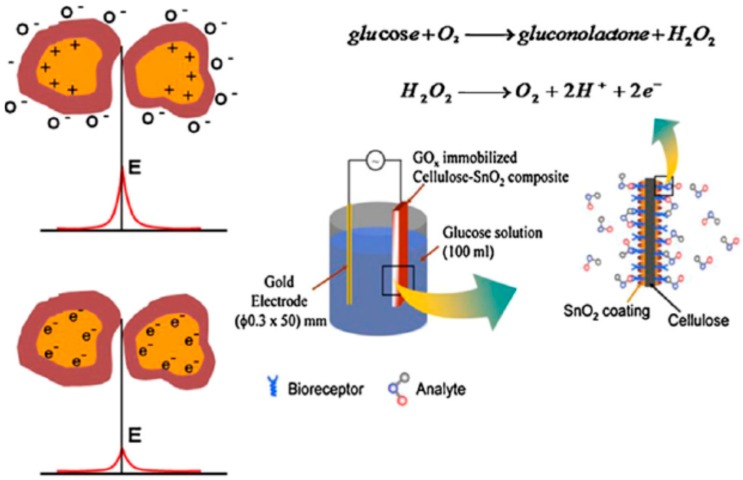
Schematic representation of the detection mechanism of a cellulose-SnO_2_ hybrid nanocomposite glucose biosensor [111].

**Figure 14:**
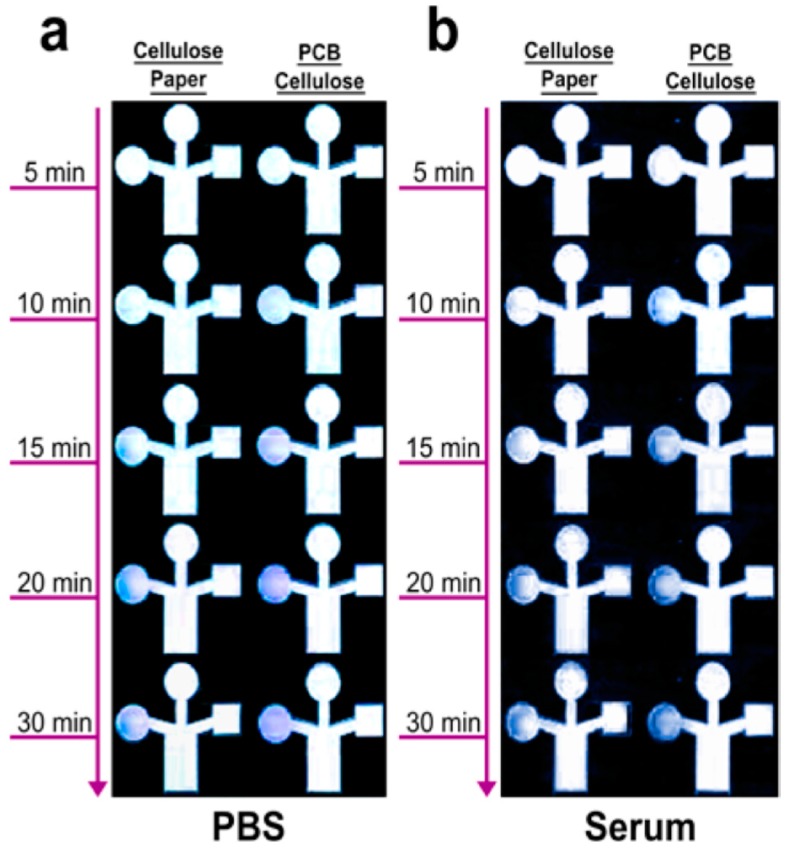
Glucose detection as a function of time using PCB modified and unmodified cellulose paper-based analytical devices. Glucose was spiked (5.0 mM) into (**a**) PBS; and (**b**) undiluted human serum [118].

Wang et al. [112] explored the potential of a gold nanoparticles-bacterial cellulose (Au-BC) nanocomposite as a platform for the amperometric determination of glucose. The detection limit for glucose under optimized conditions was as low as 2.3 mM with a linear range from 10 mM to 400 mM, and the biosensor was successfully applied to the determination of glucose in human blood samples. The compatibility of cellulose as a dielectric layer in oxide-based semiconductor thin film transistors fabricated with large-scale/large-area deposition techniques, low cost substrates, and a very low operating bias demarcates this as a promising approach to attain high-performance disposable electronic applications, such as paper displays, RFID tags, smart labels, smart packaging, and self-analysis in bio-applications [113]. Hybrid cellulose based materials with enhanced material properties for bio sensors applications have been reported [69,114]. Mahadeva et al. [111] developed a glucose biosensor based on cellulose paper, and glucose oxidase immobilized cellulose-SnO_2_ hybrid nanocomposite (Figure 13). A urea detecting sensor based on tin-oxide coated cellulose has also been studied [115], and its sensitivity was measured as a function of the urea concentration. Maniruzzaman et al. [116] investigated the possibility of using titanium dioxide (TiO_2_) for conductometric glucose biosensors by blending TiO_2_ nanoparticles into the cellulose solution to fabricate a TiO_2_-cellulose hybrid nanocomposite. The study revealed that the TiO_2_-cellulose hybrid nanocomposite can be a potential contender for cheap, disposable and flexible biosensors. A titanium dioxide (TiO_2_)-cellulose composite was fabricated by blending TiO_2_ nanoparticles with the cellulose solution and its urea biosensing behavior was studied [117]. The developed urea biosensor was highly sensitive at urea concentration lower than 10 mM and it maintained a linear response up to a urea concentration of 50 mM. Zhu et al. [118] demonstrated a polycarboxybetaine (PCB)-modified cellulose paper as a potential paper-based microfluidic diagnostic device for glucose detection from undiluted human serum (Figure 14). Due to the excellent fouling resistance and super-hydrophilic properties of PCB, devices with PCB coatings have the advantages of faster diffusion as well as a significant increase in the amount of target analytes present in the detection zone, thereby increasing their sensitivity, particularly in complex media such as undiluted human serum.

**Figure 15:**
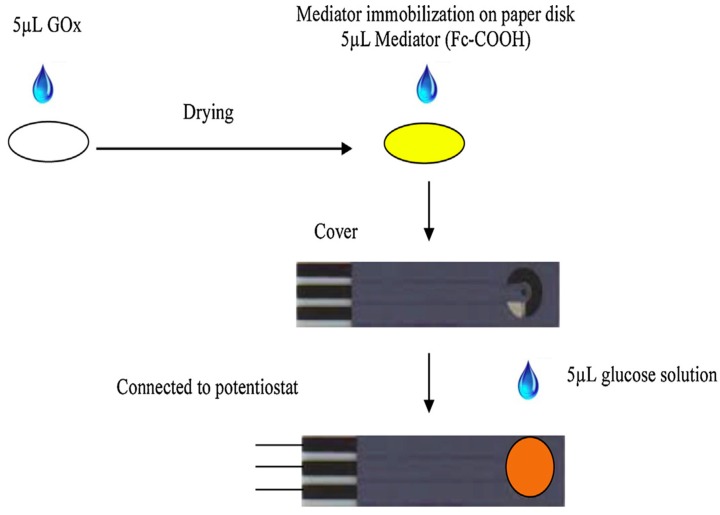
Preparation of paper disk and integration with SPCE [119].

Lawrence et al. [119] investigated a simple low cost, green biosensor configuration comprising a hydrophilic cellulose paper disk with immobilized glucose oxidase (GOx) via an adsorption step, placed on top of a screen printed carbon electrode (SPCE) (see Figure 15). Cellulose paper was also used as the pre-storage reagent matrix for 0.1 M phosphate buffer solution (PBS, pH 7.0) and 10 mM soluble ferrocene monocarboxylic acid mediator. This biosensor exhibited a linear dynamic calibration range of 1 to 5 mM glucose, with a limit of detection of 0.18 mM and retained 98% of its signal after a period of four months.

Mun et al. [120] studied the feasibility of cellulose ZnO hybrid film for a conductometric glucose biosensor. The glucose biosensor was linearly sensitive to the glucose concentration up to 12 mM. The film was able to detect glucose in the range of 1–12 mM.

### 4.4. Cellulose Based Gas and pH Sensors

**Figure 16:**
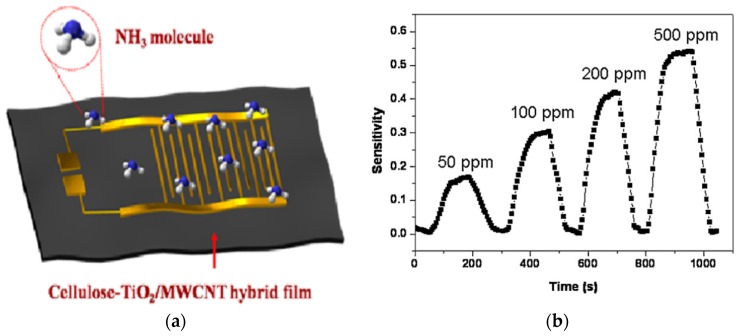
(**a**) Schematic diagram of NH_3_ CTM sensor (**b**) Sensitivity curve [124].

Cellulose based hybrid nanocomposites have also been employed for the detection of toxic and harmful gasses in gas sensors as well as chemical vapor sensors [121]. Karthigeyan et al. [122] developed highly sensitive gas sensors based on SWNT networks prepared from aqueous hydroxypropylcellulose-assisted dispersions. The sensors were capable of detecting 25 ppb or lower concentrations of NO_2_ and 5 ppm ammonia, and showed almost no baseline drift after multiple NO_2_ exposures at room temperature. Sadasivuni et al. [123] fabricated a flexible NO_2_ sensor from cellulose nanocrystal (CNC)/iron oxide composite by growing iron oxide on a nanostructured CNC sheet. The proposed sensor was capable of detecting the NO_2_ gas as low as parts-per-million in short time at room temperature. The cellulose-TiO_2_-MWNT (CTM) nanocomposite as depicted in Figure 16 showed a much higher response to NH_3_ with varying concentrations of the gas [124]. The gas sensor effectively measured traces of NH_3_ with reliability in the range of 50–500 ppm at room temperature and good sensitivity. A cellulose based sensor for detecting chlorine and nitrogen dioxide has also been reported [125]. Han et al. [126] implemented an SWNT based ammonia sensor with cellulose paper. Two types of devices were fabricated and compared: CNT-on-paper and CNT-cellulose composite. The CNT-on-paper device showed a faster response/recovery and higher sensitivity than the CNT-cellulose composite, due to the larger reaction surface. Compared to the control sensor made on a glass substrate, the paper based sensor exhibited superior uniformity and repeatability and can be utilized for smart paper based low-cost disposable applications.

Lee et al. [127] proposed a novel chemical gas sensor based on gallium nitride (GaN), a wide band gap semiconducting material used for power electronics. A gallium nitride-cellulose nanocomposite coated with inter-digit transducer (IDT) gold electrodes was characterized to make a paper like sensor for NH_3_ and NO_2_ gasses [62]. Souza et al. [128] exposed sheets of cellulosic paper modified with polyaniline nanoparticles to acidic conditions and found that the color of the modified paper sheets showed RGB color changes, as detected by a scanner. The color changes were most significant when the hydrochloric acid content was in the range of 0–500 ppm.

**Figure 17:**
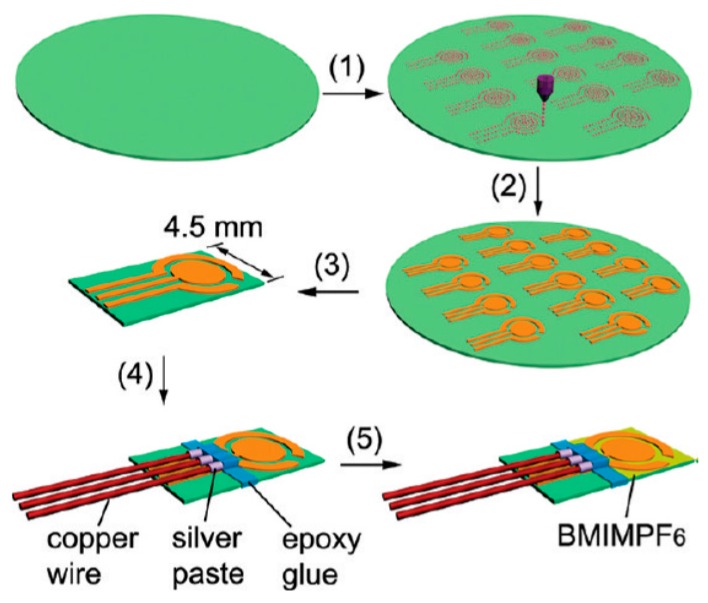
Procedures employed for the inkjet printing of gold electrode arrays on MCE membranes to fabricate paper-based solid-state electrochemical oxygen sensors: (**1**) Inkjet printing of GNP patterns; (**2**) growth of GNP patterns into gold electrode arrays; (**3**) cutting a PGEA from its ensembles; (**4**) electric connection and size control of a PGEA; and (**5**) addition of BMIMPF6 from the back MCE side of a PGEA to fabricate the oxygen sensor [132].

Mahadeva et al. [129] studied a paper-based pH sensor made of a tin oxide-cellulose hybrid composite. The comparatively good sensitivity of sensor indicates that this novel paper-based disposable sensor has the potential to be used for pH sensing applications. Gao et al. [130] reported the fabrication and characterization of a strong and transparent pH-responsive hydrogel from cellulose nanocrystals. The designed pH detector showed pronounced change in their swelling index in response to variation in pH. The reported pH responsive hydrogels could also be useful in energy storage applications due the significant improvement in the dielectric constant. Li and his coworkers [131] developed a pH-responsive shape-memory polymer nanocomposite by blending poly(ethylene glycol)-poly(ε-caprolactone)-based polyurethane (PECU) with functionalized cellulose nanocrystals (CNCs). CNCs functionalized with carboxyl groups (CNC-CO_2_H) and pyridine moieties (CNC-C_6_H_4_NO_2_) endowed the pH-responsiveness of CNCs via the association and disassociation of hydrogen bonding interactions among CNCs under different pH conditions. Hu et al. [132] established a simple approach to the mass production of nanoporous gold electrode arrays on cellulose membranes for the electrochemical sensing of oxygen using ionic liquid (IL) electrolytes (Figure 17). 

Their approach combines the inkjet printing of gold nanoparticle (GNP) patterns with the self-catalytic growth of these patterns into conducting layers, and the resulting arrays have several unique properties, including good conductivity, excellent flexibility, high integration, and low cost. This sensor, with the appearance of a piece of paper, possesses high sensitivity for O_2_ in the linear range from 0.054 to 0.177 v/v %, along with a low detection limit of 0.0075% and a short response time of less than 10 s, suggesting that it has promising applications in developing cost-effective and environment-friendly paper-based electrochemical gas sensors. Electrically conductive films composed of CNTs and a cellulose matrix were used as water sensors, and a rapid response and high sensitivity, with a relative electrical resistance change of 5500–500% for the cellulose-CNT composites with CNT loadings from 2 to 10 wt %, was observed [133].

### 4.5. Cellulose Based Paper Transistors

One of the biggest challenges in paper electronics is to produce flexible, low cost, and easily recyclable products [134]. The good characteristics of cellulose films, such as their ease of manufacturing, stability and actuation mechanism, makes them a good candidate for paper electronics devices. Its lack of intrinsic free electrons and holes impart insulating properties to cellulose.

**Figure 18:**
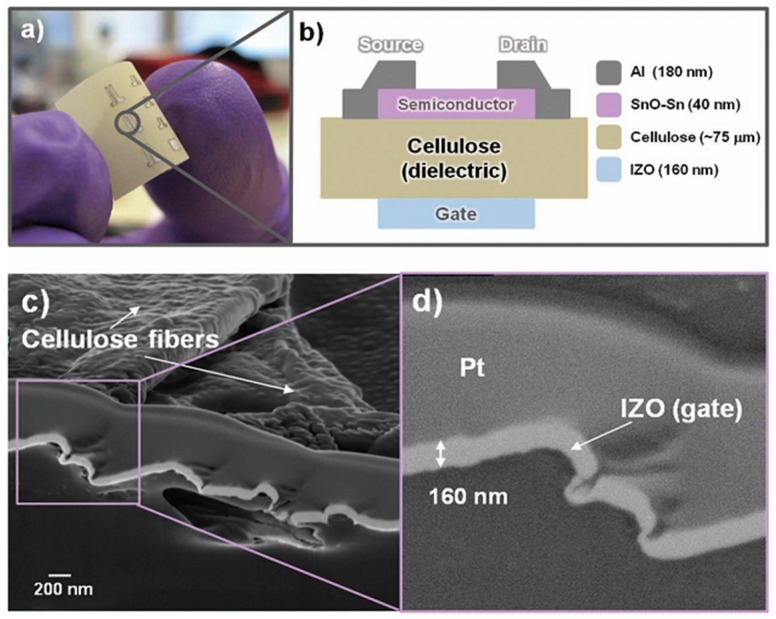
(**a**) Shows an optical photograph of a p-type paper transistor (**b**) architecture of the paper transistor (**c**) scanning electron microscopy cross section image of the gate electrode IZO [136,137] and (**d**) with a higher magnification to see the detail of the good step coverage of the oxide semiconductor over the cellulose fibers [135].

By the chemical bonding of metals or semiconductors, similar to Si doping technology, electronic channels can be formed into cellulose structures. Yun et al. [12] demonstrated a paper transistor made with covalently bonded regenerated cellulose-MWNTs (RC-MWNTs). MWNTs permit an electron channel path inside the cellulose substrate. The results confirm the potential of flexible paper based transistors for future electronics devices. Fortunato et al. [113] also reported cellulose paper based transistors for disposable electronics such as paper displays, RFIDs, and smart packaging. There was no significant difference in the electronic performance of the p-type paper transistor when the cellulose fiber was used as a dielectric [135,136,137]. The only difference was related to the on/off ratio, which is largely related to the fact that cellulose fibers have a typical open structure when limiting the off current. A schematic of the paper transistor is presented in Figure 18.

**Figure 19:**
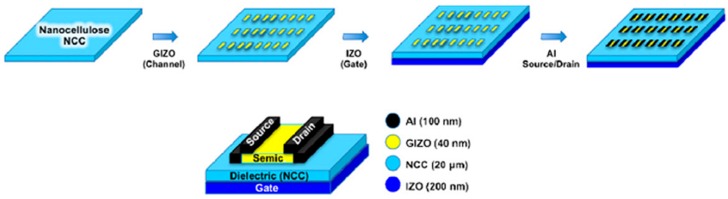
Schematics of the process of fabrication of FETs using NCC as the gate dielectric, and the corresponding staggered-bottom gate structure [140].

The cellulose nano-paper transistor showed an excellent optical transmittance of up to 83.5% and high toughness, and this device configuration can transform many semiconductor materials for use in flexible green electronics [138,139]. Gasper et al. [140] studied a cotton-based nano-crystalline cellulose, also known as nanopaper, which is a promising substrate and component for producing low cost fully recyclable flexible paper electronic devices, due to its suitable properties (lightweight, stiffness, non-toxicity, transparency, low thermal expansion, gas impermeability and improved mechanical properties). Such hybrid Field Effect Transistors (FETs) present excellent operating characteristics, such as a high channel saturation mobility drain-source, current on/off modulation ratio higher than 10^5^ enhancement n-type operation and sub-threshold gate voltage swing of 2.11 V/decade. The results showed the excellent performance of this nano-paper, making it a promising approach for high-performance disposable electronics, such as paper displays, smart labels, smart packaging, RFID (radio-frequency identification) and point-of-care systems for self-analysis in bioscience applications. Figure 19 depicts the schematics of the process of fabrication of FETs using nanocrystal cellulose (NCC) as the gate dielectric, and the corresponding staggered-bottom gate structure.

Paper substrates produced from microfibril-nanofibril cellulose has a strong influence on the performance of oxide based paper field effect transistors (FETs) [141]. It was observed that the gate leakage current in paper FETs can be reduced using a dense microfiber/nanofiber cellulose paper as the dielectric. The saturation mobility of the devices increased up to 16 cm^2^·V^−1^·s^−1^, with an I_ON_/I_OFF_ ratio close to 10^5^ by modifying the pH of the microfiber/nanofiber cellulose pulp through the addition of HCl.

### 4.6. Humidity and Temperature Sensors

Ducere et al. [142] reported on the fabrication of a robust capacitive humidity sensor manufactured using a mixture of three cellulose acetate butyrates cross-linked by a melamine formaldehyde resin as the sensor material. The sensor showed good performance from 0% to 100% RH and from −40 to 120 °C, and was robust enough to use in industrial processes. Sadasivuni et al. [143] reported a simple, flexible and partially transparent temperature sensor fabricated from composite film of reduced graphene oxide (rGO) and cellulose. The relative capacitance of the sensor changed as a function of temperature for all thermally reduced rGO filled composites. For a temperature range between 25 and 80 °C, the proposed temperature sensor exhibited a linear behavior with respect to temperature change. Kafy et al. [144] investigated cellulose nanocrystal/graphene oxide composite as humidity sensor. Performance of the composite film as a humidity sensor was evaluated on the basis of relative capacitance change at different humidity level. The proposed sensor responded linearly in log scale and its sensitivity was not affected by temperature. The resistances and capacitances of the samples fabricated by using cellulose and poly-*N*-epoxypropylecarbazole (Au/cellulose/PEPC/Au) were evaluated under the effect of humidity in the range from 30%–90% [145]. This organic humidity sensor can be used for short to long-range telemetry systems for environmental monitoring and the assessment of the humidity level. Saeed et al. [146] investigated the electrical properties of a Cu/cellulose/PEPC/Cu organic humidity sensor, in which 2 wt % of cellulose and 4 wt % of PEPC were blended in benzol. The humidity dependent properties of the sensor make it suitable to be used as a capacitive, resistive and impedance type humidity sensor for environmental monitoring. Zhang and his coworkers [147] studied the fabrication of moisture-responsive, self-standing films using cellulose stearoyl esters (CSEs) with diverse degrees of substitution (DSs). The films of CSE with a low DS of 0.3 (CSE_0.3_) exhibited moisture-responsive properties, whereas CSEs with higher DSs of 1.3 or 3 (CSE_1.3_ and CSE_3_) did not. It was shown that within a local moisture gradient, the CSE_0.3_ films could reversibly fold and unfold as rhythmical bending motions due to the absorption and desorption of water molecules at the film surface. Furthermore, a combined responsiveness to moisture and temperature was exhibited by bilayer films containing one layer of CSE_0.3_ at one side and one layer of CSE_3_ at the other side.

**Figure 20:**
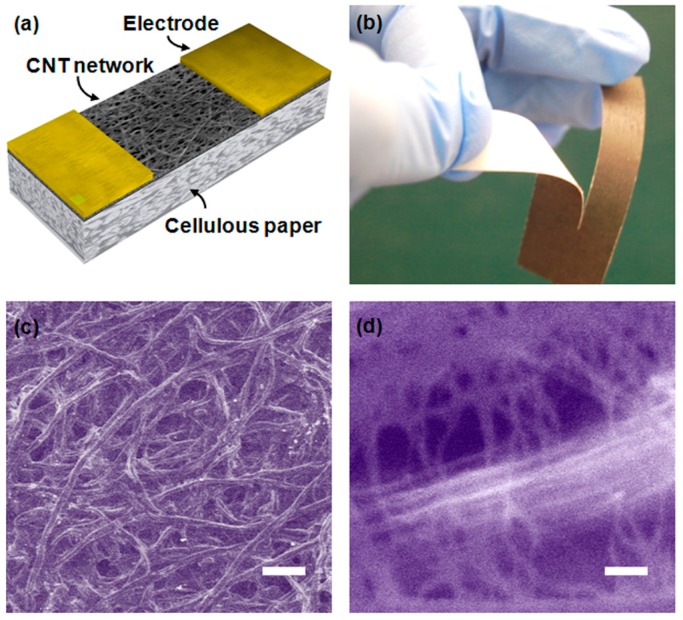
(**a**) Schematic illustration of the humidity sensor built on a cellulose paper substrate; (**b**) networks of CNTs on the paper showing that the device is flexible and custom-cut; (**c**) SEM image of the cellulose paper (scale bar 250 μm); and (**d**) magnified image of the cross-linked CNTs (scale bar 100 nm) [148].

Han et al. [148] demonstrated a humidity sensor on a cellulose paper using single-walled carbon nanotubes functionalized with carboxylic acid. As compared to the sensor made on a glass substrate, the cellulose mediated charge transport on the paper substrate enhances the sensitivity and provides a breakthrough towards future paper electronics for low cost disposable applications (Figure 20).

Lokman et al. [149] suggested a humidity sensor based on a tapered single-mode fiber coated with a mixed hydroxyethyl cellulose/PVDF polymer composite. The performance of the sensor was calculated for two fiber diameters, 50 and 87.5 μm. As the relative humidity increases, the interference spectrum shifts towards longer wavelengths. The highest sensitivity, 0.0116 nm/%, was obtained at the smallest tapered-fiber diameter of 50 μm, with the linearity being more than 98.20%.

**Figure 21:**
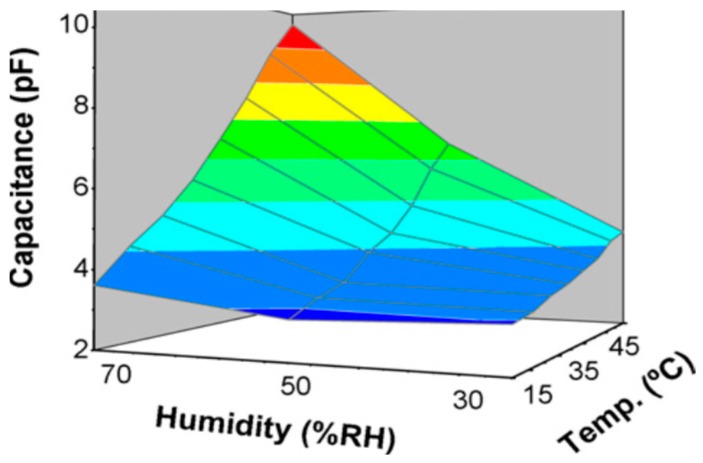
Capacitance of cellulose-PPy nanocomposite sensor (CP-16) as a function of temperature and humidity [150].

Mahadeva et al. [150] introduced a cellulose-polypyrrole (PPy) nanocomposite by coating a nanolayer on a cellulose membrane under varying polymerization times. Nanoscaled PPy was established onto a cellulose surface via an in-situ polymerization without disturbing the cellulose structure. The capacitance of the humidity sensors changes with increasing value of the humidity. Due to its chemical structure, cellulose is sensitive to humidity in the air. The adsorption and desorption of water molecules show good repeatability as shown in Figure 21.

### 4.7. Acoustic Applications

**Figure 22:**
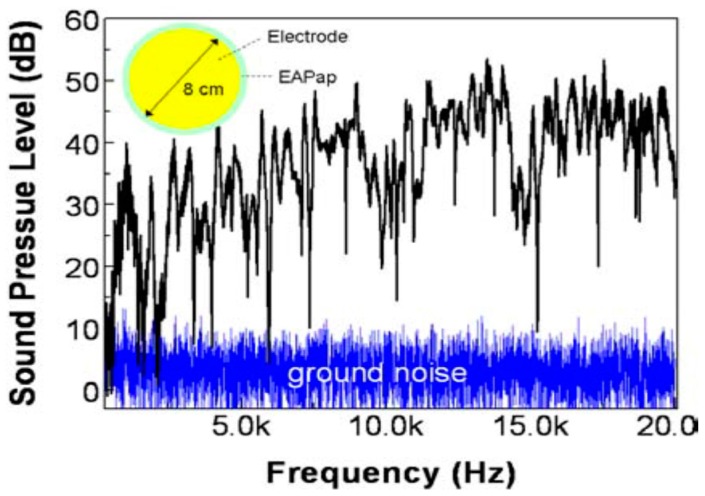
The acoustic performance of the circular plate EAPap speaker. A maximum sound pressure of up to 53 dB was achieved at 13 kHz. The inset shows the electrode size on the piezoelectric cellulose EAPap film [152].

Transparent thin film acoustic transducers based on a PVDF thin film coated with compliant CNTs were effective both as acoustic actuators (speakers) and sensors (microphones) [151]. The increased piezoelectricity of cellulose EAPap due to mechanical stretching proved that the flexible regenerated piezoelectric cellulose can be applied to acoustic applications such as thin piezoelectric paper speakers [11]. Kim et al. [130] characterized a film type flexible cellulose-EAPap speaker in the audible range considering the piezoelectricity of EAPap and the performance of the EAPap speaker was comparable to that of a PVDF piezoelectric thin film. The piezoelectric behavior of the thin EAPap surface was visualized at 6 kHz and 221 Hz (see Figure 22). The developed cellulose based transducers could make a significant impact on room acoustics by enabling windows, touch panels, and computer screens to act as invisible speakers and microphones.

## 5. Conclusions

In this paper, we have reviewed the recent progress of cellulose-based electro-active paper and its hybrid nanocomposite application devices. Cellulose has been investigated as a smart material that can be used as biomimetic actuators and sensors. Cellulose hybrid nanocomposites made by coating a conductive polymer and SWNTs/MWNTs with cellulose improved the displacement output and force output of EAPap actuators. An integrated overview of the fabrication techniques, environmental effects, material properties and working principle of cellulose EAPap is also presented. Since cellulose EAPap materials are biocompatible, sustainable, biodegradable, capable of broad chemical modification, hydrophilic, high mechanical strength and stiffness, and have an interesting actuation mechanism, a large number of cellulose based devices are possible.

Due to the interesting actuation mechanism of cellulose, which is a combination of ion migration and the piezoelectric effect, and its smart characteristics, cellulose EAPap has been applied to haptic actuators, remotely controlled and powered actuators, actuators for micro-robots and flying objects. The enhanced piezoelectricity of EAPap allows it to be used in strain sensors, flexible film type speakers for acoustic applications, energy harvesting from ambient vibration, and self-powered vibration sensors for structural health monitoring.

Cellulose-based humidity and temperature sensors, as well as paper transistors for paper electronics applications, have also been reported. Among the many other attractive applications of cellulose, nature friendly, cost-effective, metal oxide-cellulose hybrid nanocomposite biosensors and chemical disposable sensors with good sensing performance are discussed. Various types of cellulose-based EAPap and its potential applications are summarized in Table 1. There are many possibilities to use cellulose EAPap as a smart material, and cellulose hybrid nanocomposites have high potential.

However, more research into cellulose based hybrid materials is required to develop real world applications that are cheap, safe, stable in a harsh environment, accurate and can be mass produced. Considering the current integration technology, cellulose based paper biomimetic actuators and sensors have great potential for cheap, disposable and biocompatible devices, with recycling also being available after use, although some challenges still remain in terms of the material robustness, reliability, stability in harsh environments, and niche application development.

## Figures and Tables

**Table 1:** Summary of cellulose-based composites, type of blend/coating, chemical nature (organic/inorganic) and potential applications.

No.	Name	Type of Blend/Coating	Chemical Nature of Coating/Blend	Applications	Ref.
1	Conducting polymer and SWNT coated cellulose EAPap	Composite of SWNT/polyaniline with dopants (${\text{Cl}}^{-}\;\mathrm{and}\;{\text{ClO}}_{4}^{-}$)	Inorganic	Ultra-light weight smart actuator	[45]
2	MWNTs mixed cellulose EAPap	MWNTs	Inorganic	Bending EAPap actuator	[46]
3	Functionalized-MWNTs blended cellulose EAPap	Functionalized-MWNTs	Inorganic	Micro-robot, micro-flying objects ,sensors	[47]
4	Aligned MWNT/Cellulose composite	MWCNTs covalently grafted to cellulose	Inorganic	Actuator	[48]
5	TiO_2_/MWNT/Cellulose hybrid nanocomposite	TiO_2_/MWCNTs	Inorganic	pH sensors	[49]
6	Biocompatible SWNTs/Cellulose composite	SWNTs	Inorganic	Cell sensors	[50]
7	SWNTs bonded cellulose composite	SWNTs	Inorganic	Flexible paper transistors	[51]
8	Cellulose-chitosan laminated films as EAPap	Chitosan with free ions (${\text{Cl}}^{-},\;{\text{NO}}_{3}^{-}$ and ${\text{CF}}_{3}\;{\text{COO}}^{-}$)	Organic-inorganic composite	Humidity-less-sensitive EAPap actuator	[55]
9	Chitosan-blended cellulose EAPap	Chitosan	Organic	Blood clotting patches, artificial muscle, biomimetic pump	[56]
10	Cellulose derivative composite	hydroxypropylcellulose (HPC) and acetoxypropylcellulose (APC)	Organic	Electro-optical sensors	[68]
11	TiO_2_-cellulose nanocomposite mixed cellulosic fibers	TiO_2_ particles	Inorganic	Highly opaque papers	[69]
12	SnO_2_ nanoparticle loaded cellulose hybrid thin films	SnO_2_ nanoparticles	Inorganic	Low cost, flexible and biodegradable humidity sensors	[70]
13	Cellulose/silica and silica-gold hybrid biomaterials	Silica and Silica-gold particles	Inorganic	Electronics applications	[63]
14	LiCl/Cellulose EAPap	LiCl content	Inorganic	Humidity-less-sensitive EAPap actuator	[82]
15	Polyaniline-coated EAPap	Polyaniline-coating	Organic	actuators	[83]
16	Sodium alginate/cellulose EAPap	Sodium alginate	Organic	Humidity-less-sensitive EAPap actuator	[84]
17	Ionic liquid blended cellulose EAPap	Ionic liquids (BMIPF_6_, BMICL, BMIBF_4_)	Inorganic	Durable humidity-less-sensitive EAPap actuator	[94,95,96]
18	Cellulose acetate double membrane actuator	Cellulose acetate layers	Organic	Kinesthetic actutors for haptic devices	[99]
19	Cellulose-polypyrrole-ionic liquid nanocomposite	Polypyrrole-ionic liquid	Organic	EAPap actuators	[100]
20	Cellulose-polypyrrole-ionic liquid EAPap	Polypyrrole-ionic liquid	Organic	Biomimetic robots, remotely driven actuators, remote sensing units, portable electronics	[101]
21	Cellulose EAPap coated with gold electrodes	Gold electrodes	Inorganic	Electromechanical energy harvesting transducer	[105]
22	Gold nanorods/cellulose acetate composite film based biosensor	Gold nanorods	Inorganic	Amperometric glucose bisensor	[109]
23	Gold nanoparticles-bacterial cellulose nanocomposite	Gold nanoparticles-bacteria	Inorganic-organic composite	Biosensor for determination of glucose in human blood	[112]
24	Cellulose/tin oxide hybrid nanocomposite	Glucose oxidase (GO_x_)/ tin oxide (SnO_2_)	Organic-inorganic composite	Glucose biosensor	[111]
25	Tin-oxide coated cellulose	Porous tin-oxide (SnO_2_)	Inorganic	Urea detecting sensor	[115]
26	TiO_2_-cellulose hybrid nano composite	TiO_2_ nanoparticles	Inorganic	Conductometric glucose biosensor	[116]
27	Polycarboxybetaine functionalized cellulose paper	Polycarboxybetaine	Organic	glucose detection from undiluted human serum	[118]
28	Hydrophilic cellulose paper disk with immobilised glucose oxidase	Glucose oxidase (GO_x_)	Organic	Food processing control, biotechnological analytical devices	[119]
29	MWNTs-cellulose paper	MWNTs	Inorganic	chemical vapor sensor	[121]
30	SWNT-network-based gas sensors	SWNTs networks	Inorganic	Room-temperature gas sensors	[122]
31	Cellulose-TiO_2_-MWNT nanocomposite	TiO_2_-MWNTs	Inorganic	NH_3_ sensor	[124]
32	CNT-on-paper, CNT-cellulose composite	SWNTs	Inorganic	Ammonia sensor	[126]
33	Gallium nitride-coated cellulose nanocomposite	Gallium nitride	Inorganic	NH_3_ and NO_2_ gas sensor	[62]
34	Cellulose Paper Sheets with Polyaniline Nanoparticles	Polyaniline Nanoparticles	Organic	Acid concentration sensor	[128]
35	Tin oxide-cellulose hybrid composite	Tin-oxide	Inorganic	pH sensor	[129]
36	Nanoporous gold electrode arrays on cellulose membranes using ionic liquid electrolytes	Gold, ionic liquid	Inorganic	Electrochemical Oxygen Sensors	[132]
37	Cellulose-CNTs composite	CNTs	Inorganic	Water sensors	[133]
38	Regenerated cellulose-MWNTs flexible paper	MWNTs	Inorganic	Flexible paper transistor	[12]
39	Cellulose acetate butyrate cross-linked by a melamine formaldehyde resin	Melamine formaldehyde resin	Organic	Humidity and temperature sensor	[142]
40	Cellulose and poly-N-epoxypropyl-carbazole	Poly-N-epoxypropylcarbazole	Organic	Humidity sensor	[145]
41	Cellulose with carboxylic acid functionalized SWNTs	Carboxylic acid functionalized SWNTs	Inorganic	Resistor-type humidity sensors	[148]
42	Cellulose-polypyrrole nanocomposite	Nanoscaled polypyrrole (PPy)	Organic	Capacitive-type humidity and temperature sensor	[150]
43	PVDF thin film coated with compliant CNTs	CNTs	Inorganic	Acoustic actuators (speakers) and sensors (microphones)	[151]
44	Cellulose/BaTiO_3_ paper	BaTiO_3_	Inorganic	Sensing devices	[72]
45	Cellulose/graphene nanocomposite	Functionalized graphene oxide	Inorganic	Disposable solvent sensor	[75]
46	Hybrid thin film of graphene nanoplatelets and cellulose nanocrystals	Graphene nanoplatelets	Inorganic	Packaging, electrical and heat conducting applications	[80]
47	TiO_2_-Cellulose composite	TiO_2_	Inorganic	Urea biosensing	[117]
48	Cellulose nanocrystal/iron oxide composite	Iron oxide	Inorganic	Flexible NO_2_ sensor	[123]
49	Cellulose/reduced graphene oxide composite	Reduced graphene oxide	Inorganic	Temperature sensor	[143]

## Abbreviations

The following abbreviations are used in this manuscript:

PVDF	polyvinylidene fluoride
EAPap	electro-active paper
SWNT	single walled carbon nanotube
MWNT	multi walled carbon nanotube
PEO-PEG	poly(ethylene oxide)-poly(ethylene glycol)
CNT	carbon nanotube
CPIL	cellulose-polypyrrole-ionic liquid
PPy	polypyrrole
PCB	poly carboxybetaine
CTM	cellulose-TiO_2_-MWNT
SPCE	screen printed carbon electrode
IDT	inter-digit transducer
IL	ionic liquid
GNP	gold nanoparticle
RFID	radio-frequency identification
NCC	nanocrystal cellulose
CNC	cellulose nanocrystals
GnP	graphene nanoplatelets
CMC	carboxymethylcellulose
